# A Review of Rodent Behavior, Mobility, and Pain Modifications in Response to Destabilization of the Medial Meniscus Injury

**DOI:** 10.3390/biomedicines13122886

**Published:** 2025-11-26

**Authors:** Heidi Kloser, Marcela Henao-Tamayo, Kelly S. Santangelo

**Affiliations:** Department of Microbiology, Immunology, and Pathology, Colorado State University, Fort Collins, CO 80523, USA; heidi.kloser@colostate.edu (H.K.); marcela.henao_tamayo@colostate.edu (M.H.-T.)

**Keywords:** osteoarthritis pain, behavior and mobility, destabilization of the medial meniscus (DMM), OA rodent models, open field testing, gait analysis, mechanical allodynia, knee pain

## Abstract

Increasing emphasis is being placed on evaluating pain and mobility outcomes of osteoarthritis (OA) in both clinical and preclinical studies. In rodent models of post-traumatic OA (PTOA), particularly those utilizing destabilization of the medial meniscus (DMM), behavioral assays are becoming more prominent as researchers seek to bridge the translational gap between structural joint pathology and human disability. However, substantial variability exists in how behavior, mobility, and pain are assessed, potentially limiting reproducibility and cross-study comparisons. This review evaluates the current literature on behavioral and pain-related outcomes in rodent DMM models, with the aim of cataloging observed phenotypes, identifying methodological inconsistencies, and proposing recommendations for standardization. We compiled data on a range of behavioral assays, including mechanical and thermal sensitivity, cage monitoring, gait analysis, weight distribution, balance, and joint compression, to construct a temporal framework of post-injury changes in behavior. Across studies, behavioral changes were observed as early as one day post-injury and persisted up to 30 weeks, with notable variability depending on age, sex, and testing protocols. Young male mice (≤12 weeks old) were the most extensively studied. The findings highlight clear trends in pain sensitivity and functional decline but also underscore the need for harmonized methodologies and reporting standards. By providing a comprehensive synthesis of behavioral outcomes post-DMM, this review aims to support more informed study design and interpretation, offering a foundation for greater consistency and translational relevance in future rodent DMM research.

## 1. Introduction

Sonya Glasson and Elizabeth Morris first introduced the destabilization of the medial meniscus (DMM) rodent injury model between 2004 and 2007 as a new and more reliable method for studying osteoarthritis (OA) and post-traumatic OA (PTOA) in small animal models [1,2,3,4,5]. This technique was spurred by the need to develop a model that more closely mimicked the disease progression observed in larger animal models and humans. In this method, the meniscotibial ligament of the medial meniscus is transected from the medial meniscus to create an unstable environment and induce OA [1,6]. Glasson et al. initially pioneered this method in mice and, since then, DMM has expanded to other species [1].

OA is a progressive, multifactorial joint disorder estimated to affect over 595 million people worldwide as of 2020 [7]. Risk factors for OA include age, female sex, joint trauma, obesity, and genetics [8,9]. OA is characterized by cartilage degradation, synovial inflammation, and subchondral bone remodeling [10,11,12,13]. It leads to pain, stiffness, and loss of mobility, contributing to physical disability, economic burden, and decreased quality of life [7,11,14,15,16,17,18]. Globally, as of 2023, there is an estimated 300 billion dollars spent on OA-related health care costs annually. In the US alone, the economic burden is expected to reach 6.4 billion dollars each year [16,19]. Treatments remain primarily aimed at symptom modification (analgesics including non-steroidal anti-inflammatory drugs (NSAIDs), intra-articular therapies, assistive measures, and ultimately joint replacement for end-stage disease). Currently, there is no established cure or widely available disease-modifying therapy that reliably halts or reverses cartilage loss [7,20,21,22]. Because a substantial proportion of clinically important OA arises after joint injury, there is intense interest in understanding PTOA mechanisms and in developing therapies that preserve structure and function [9,23,24].

At the time Glasson and Morris introduced the DMM model, OA was commonly induced by collagenase injections, anterior cruciate ligament transection (ACLT), or studied in spontaneous OA models (transgenic and aged) [1]. Results from collagenase injections were inconsistent or demonstrated variability [1,2]. In some cases, bacterial collagenase injections can lead to collagenase-mediated destruction of multiple ligaments, resulting in severe joint instability as soon as 1 day after injection or at varying times after injection [1,25]. Other joint degeneration resulting from collagenase was attributed to bone deterioration [1]. Regardless of the type of joint deterioration, there were concerns regarding the translation of collagenase to naturally occurring OA [1,2].

ACLT surgery is a technical surgery and can lead to very rapid and severe PTOA pathology and pain, particularly in mice [1,26,27]. The accelerated development of disease might restrict an opportunity to study the mechanisms of PTOA, as maximal pathology scores are most often reached at 4 weeks post-injury [1]. Additionally, this rapid deterioration of the joint may be considered less clinically relevant than a method that produces slower-progressing PTOA [1].

Spontaneous OA models, generated via transgenic mice or aged animals, are often less desirable due to the high monetary and time-related costs associated with their creation, as well as the reliance on specific genetic backgrounds [1,8,28]. While aged and naturally occurring models are relevant for studying both histological and pain-related outcomes, the OA onset is slow, and there is increased variability compared to surgical models [1,29,30].

Given this, Glasson and Morris had ample motivation to find a model that could more closely imitate OA in humans and larger animals. With results from unpublished guinea pig studies demonstrating the ease and consistency of this new method [1], they set out to determine if the DMM technique in the mouse could address some of the unmet needs in the field. They found that DMM surgery was easier to perform and resulted in a slower progression of PTOA, with differences between DMM and sham-operated animals observed at 4 weeks and increasing in severity until the study’s conclusion at 8 weeks [1]. Since then, this model has become a reproducible and widely used animal model for studying PTOA, with a slower progression of joint degeneration compared to other models.

Mice and rats are both commonly used in OA studies as they are both affordable and easy to work with, with mice being more commonly used than rats, especially as they are the primary species for transgenic models [8,11,22,31,32]. Numerous factors have contributed to mice becoming the primary species for DMM studies, and their use is extensively documented in the literature. In contrast, rats and other species are far less studied; among the 102 studies surveyed here, only 13 involved rats [22,31,33]. Each of these species differs in practical and biological ways (disease time course, sensitivity to injury, and behavioral readouts, weight bearing, and gait differences); thus, outcomes are not always directly translatable across species, and a full review could be devoted to the species-specific differences between mice and rats [8,11,12,31,34].

Similarly, sex and age markedly influence OA susceptibility and phenotype [2,11,35]. Female rodents often show different disease trajectories than males, with males having accelerated joint degeneration over females [6,11,36,37,38,39]; studies show that ovariectomies accelerate disease progression in females, implicating sex-hormone effects [11,40]. Additionally, studies have documented that, in humans and rodents, females experience greater levels of pain and are more sensitive to pain than males, and, as such, decreased histology scores have been associated with protective pain [11,41,42]. Conversely, studies have historically used largely male cohorts, which can bias histological scoring and the interpretation of results [2,35,38,43,44,45]. Increased age has also been shown to accelerate the progression of OA in rodent models, as aged animals spontaneously develop OA and are used as naturally occurring OA models; age is also one of the primary risk factors for OA in humans [9,11,18,39,46,47,48]. Selecting appropriate species, matching animals by sex and age, and using relevant structural, behavioral, pain, and mobility assays are critical for translating preclinical findings to human OA biology and therapy.

In the C57BL/6 background male mice (the most common strain for studying these changes in the reviewed articles), significant changes in joint pathology from the DMM-injured animals to the sham control animals are observed as early as 1 to 2 weeks post-injury (PI), with structural modifications worsening progressively over time [46]. In the early time points, PI, synovial inflammation, and hyperplasia with increased synovitis scores are the predominant changes observed in the knee joint. More overt cartilage degradation and osteophyte formation typically occur at later time points (4–8 weeks and beyond) [39,41,49,50,51]. Age can accelerate the severity of these changes, as it can also be a driving force for OA [18,29,46,50,52,53]. Structural modification in the joint tends to follow a similar progression pattern (as above) in aged male mice, when compared to age-matched controls; however, older animals commonly develop a greater magnitude of cartilage damage even when the temporal staging is similar [29,50,54].

Interestingly, female mice exhibit considerably different joint pathology from male mice [2,29]. Pathological differences are not observed until 4 weeks PI in the female animals. Cartilage matrix depletion is typically the first change observed in some mice; however, statistically significant scores are not typically observed until week 8 PI. Additionally, at week 8 PI, vertical fissures into the deeper cartilage zones and delamination of the superficial cartilage layer are seen. By week 12 PI, joint degeneration is progressively worse, and severe cartilage erosion and exposed subchondral bone are typically found [38,55]. Regarding other rodent species, the progression of the DMM injury in rats is also slow, with disease progression in males occurring on a similar timeline as that observed in male mice [56,57,58]. In female rats, differences in joint pathology scores are seen as early as week 8 PI [59].

**Figure 1:**
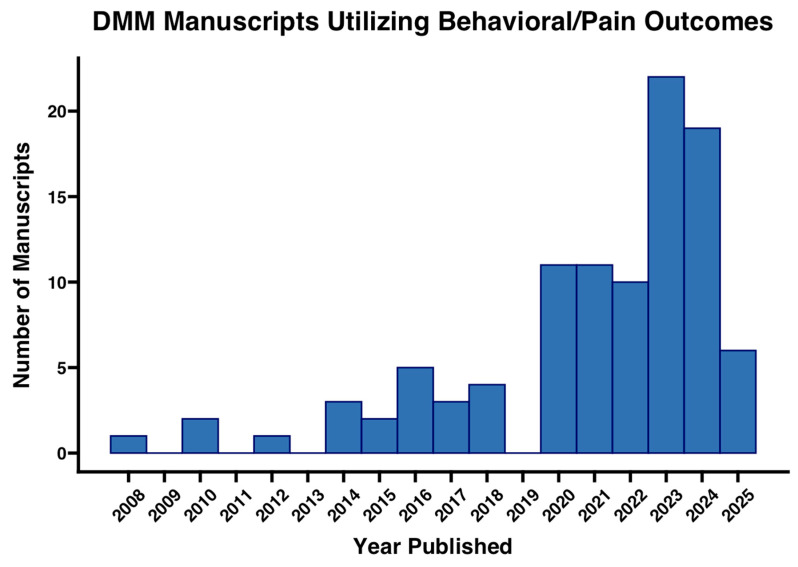
DMM manuscripts utilizing behavioral/pain outcomes in rodents. A histogram depicting the number of manuscripts including at least one behavioral/pain outcome measure since the establishment of the model in 2008 to 31 January 2025. Of note, this only includes one month for 2025; thus, this number is likely on track to exceed 2024.

As the DMM model has become a cornerstone for studying PTOA and evaluating potential therapeutics, researchers have increasingly integrated assessments of pain, behavior, and functional mobility into their study designs [43,60,61]. While early DMM studies primarily focused on histopathological and structural outcomes, there has been a notable increase in publications incorporating behavioral and pain-related outcomes (Figure 1). This shift reflects a growing recognition that, in human OA, alleviating pain and restoring mobility are paramount clinical priorities [6,40,43,55,62,63,64,65]. Additionally, research in human and animal studies has shown that outcomes from pain tests may be more indicative of the pain state or a predicted pain state than histological or radiographic severity [42]. In response, funding agencies such as the NIH have emphasized the importance of pain research, while advancements in behavioral monitoring technologies have made these assessments more accessible and cost-effective [66].

Despite this progress, the field currently lacks a consolidated resource that synthesizes the assays being used, when they are applied, and how behavioral outcomes evolve in relation to disease progression. This comprehensive review addresses that gap by comparing and consolidating findings from DMM studies on pain/behavior and functional assessments in rodent DMM models across three primary categories: evoked measures, spontaneous/compulsory measures, and other functional assessments. Within each category, we systematically analyze each of the assays, the methodologies employed, quantify the consistency of reported outcomes (highlighting key consistencies and discrepancies across studies), and establish critical timetables showing when significant behavioral differences emerge between DMM-injured animals and various control groups (baseline, sham, and naïve cohorts). The ultimate purpose of this review is to clarify the current state of behavioral assessment in DMM research, provide expected timetables for significant behavioral difference onset, and support the development of more effective and translationally relevant study designs moving forward.

## 2. Inclusion/Exclusion Criteria

The authors conducted a comprehensive review to provide a broad overview of the current methods used to assess pain and mobility/behavioral changes in the DMM injury, to provide timetables of when these changes are expected to occur, and to provide guidelines for improving pain and behavior studies in the OA field. Original research articles reviewed in this review were identified via a PubMed search for ((destabilization [Title/Abstract] AND “medial meniscus” [Title/Abstract]) OR “DMM” [Title/Abstract]) AND (rodents [Title/Abstract] OR rat [Title/Abstract] OR mouse [Title/Abstract] OR mice [Title/Abstract]) AND (behavior OR mobility OR (“overhead” AND “cage” AND “monitoring”) OR pain OR gait) AND (journal article [Publication Type] NOT review [Publication Type]) Filters English on 31 January 2025. This search yielded 219 results spanning the years 1983 to 2025. Once identified, abstracts were scanned for relevance to the field, excluding any articles that matched keywords but were not OA DMM studies, and did not include any behavioral or pain analyses. Additionally, 17 articles that fit the criteria and included OA and DMM were excluded via a quality control check, for criteria such as: not including baselines or controls; the conclusions not matching the shown data; the scope of the study included OA but was not the outcome measured; an absence of showing pain or behavior measures; a discussion of pain/behavior but not including tests or results; statistics not included in comparisons between controls and untreated DMM animals; the inclusion of DMM injury only when paired with another injury; and/or not including information on age, sex, and strain. As it was variably mentioned in methods, the inclusion of blinding was not considered as a factor to include or exclude original research articles at this stage. The authors did their best to eliminate bias in the search selection and exclusion of articles, but with the exclusion of articles, there is always a chance for bias. The authors also included one additional article that did not appear in the original PubMed search and was suggested by a reviewer, which otherwise fit the search criteria. Once sorted, the results equated to 102 original research articles cited in this manuscript (Supplementary Table S1).

## 3. Discussion–Mice

**Figure 2:**
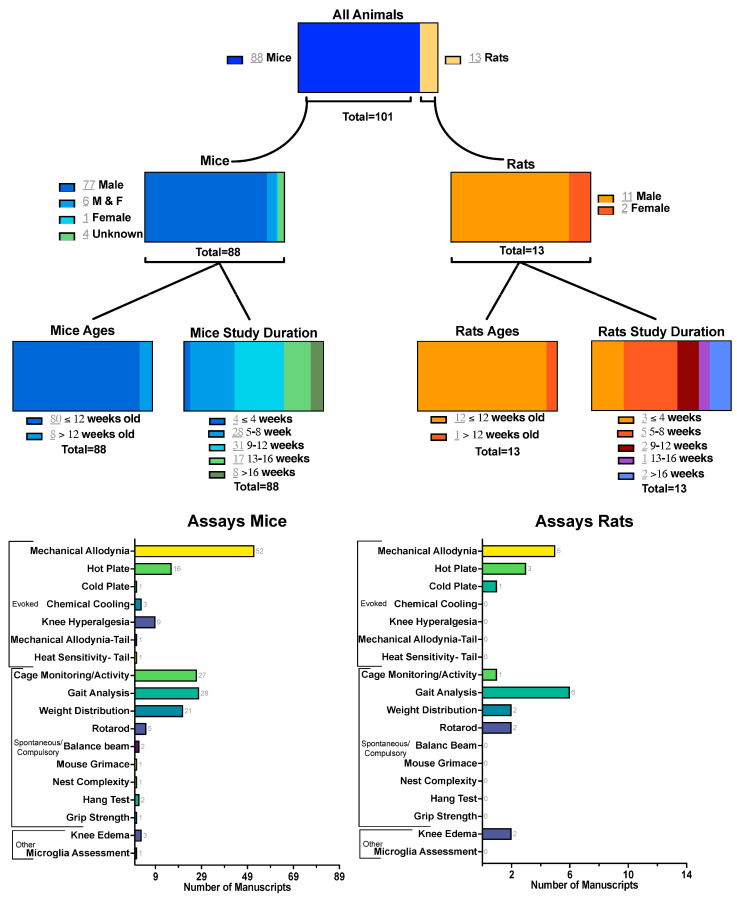
Demography of the reviewed studies. Most studies utilized male mice 12 weeks of age or younger at the time of the DMM injury. There was no clear preference on study duration for either mice or rats. The majority of the mouse studies focused on pain behavior from mechanical allodynia, and many rat studies used gait measures as their primary outcome of pain or functional mobility. Many of the studies included assessments of multiple outcomes; this figure shows the total number of manuscripts that utilized each measure. Totals are shown as grey numbers.

Of the 102 research articles included in this review, the vast majority (88 studies) used mouse models, while only 13 utilized rats (notably, both mouse and rat studies used a variety of strains) (Figure 2 and Supplementary Table S1). This distribution reflects the widespread reliance on mice in DMM studies, likely due to the availability of genetic tools and established protocols [67]. Given that the DMM injury tends to produce a more consistent and severe pathology in male rodents [2,6], most studies (94 studies) considered male animals, with only 9 incorporating females and four studies that did not specify sex. The age at the time of injury was also fairly consistent among the reviewed studies, with most studies (93 studies) using animals 12 weeks old or younger. Notably, mechanical allodynia testing emerged as the most frequently employed behavioral assay in mouse studies (52 studies), while gait analysis was the most common approach in rat models (6 studies). These trends highlight both the methodological preferences in the field and potential gaps in sex and age representation that merit further attention.

### 3.1. Evoked Measures

This section discusses assays that utilize evoked pain responses, where evoked pain is experimentally applied sensory stimulation that provokes a measurable nocifensive response and is used to probe peripheral and central sensitization in osteoarthritis and related models [22,43,68]. In this section, the evoked assays addressed include mechanical sensitivity tests (most commonly von Frey filament testing of the hind paw, assessing referred mechanical allodynia, and direct knee hyperalgesia measures using pressure-application devices), and thermal sensitivity tests (such as hot- and cold-plate tests, and chemical cooling). In translational work, pressure-evoked knee stimulation has been applied with fMRI-compatible devices to map stimulus-evoked nociceptive circuitry that is largely separable from the prefrontal–limbic representations of spontaneous chronic pain, highlighting that evoked assays primarily probe acute nociceptive pathways distinct from affective components of ongoing pain [68]. These tests are direct and indirect measures of joint pain: they reveal increased limb sensitivity or hyperalgesia via reflexive withdrawal, or vocalization, rather than capturing spontaneous ongoing pain states [43,68,69]. Below, each subsection provides a brief background on how the specific assay fits within the evoked pain category.

#### 3.1.1. Mechanical Allodynia

**Figure 3:**
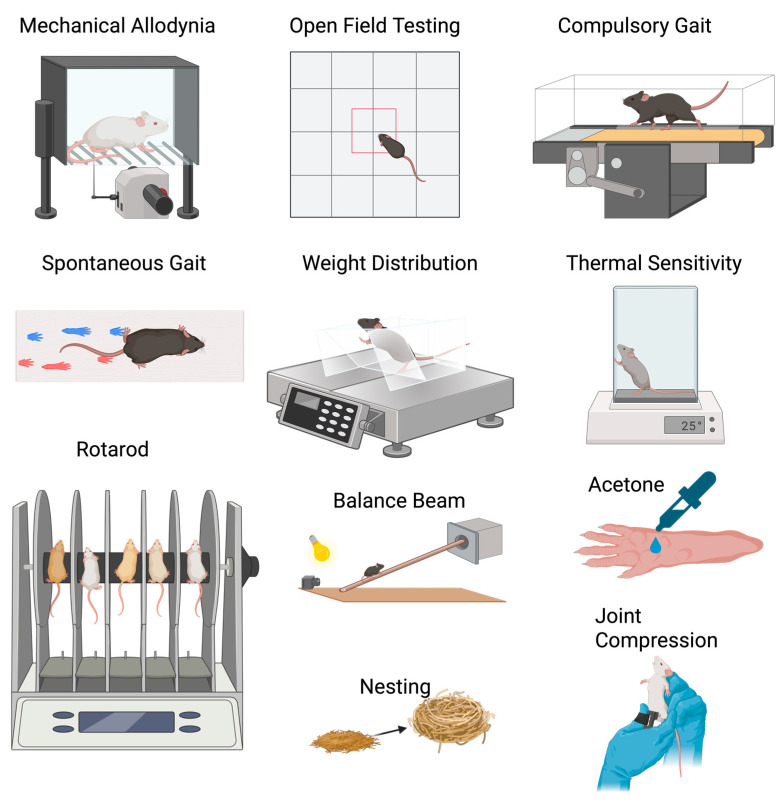
Diagram of assays. The schematic illustrates cartoon depictions of assays used in the reviewed manuscripts. Created in BioRender. Henao-Tamayo, M. (2025) https://BioRender.com/1jy6am0.

Mechanical allodynia tests measure evoked pain using mechanical stimuli (controlled physical pressures or forces applied to the paw, skin, joint, or other tactile area) [62] (Figure 3). These tests are indirect measures of knee pain that demonstrate increased limb sensitivity or overall hyperalgesia on an injured limb. They test sensitivity by measuring how much force or irritation the animal is willing to tolerate before it withdraws (lifts, flicks, licks, or flinches) its paw, or jumps [10,15,16,21,24,37,38,39,40,41,47,48,49,50,51,54,55,61,67,70,71,72,73,74,75,76,77,78,79,80,81,82,83,84,85,86,87,88,89,90,91,92,93,94,95,96,97,98,99,100,101]. A human physiologist, Maximilian von Frey, developed the first type of this test in the 1890s. He utilized these flexible, thin, and sharp fibers to locate pain-sensitive spots on human skin and pioneered the understanding of pain and pain testing [102,103]. As they were described first, it is not surprising that, to this day, the von Frey tests are the most used mechanical allodynia tests, appearing in a total of 52 manuscripts, with only one manuscript opting for different mechanical allodynia tests [76].

Von Frey assays can be performed using the traditional/manual method, which involves a set of flexible filaments with increasing strength and is scored manually [47,81]; or via an electronic von Frey Anesthesiometer, which utilizes increasing force and automatically detects withdrawal [16,78]. Both manual and electronic methods assess the same principle or research question, to test sensitivity and measure response thresholds; however, the collections and calculations differ slightly. The differences in methods and analysis can lead to variability between various studies. As such, it is essential to acknowledge these differences and limitations to accurately interpret the findings and design suitable experiments.

The responses measured by these papers fit into three method/analysis categories. The first and most frequently utilized method, with 42 manuscripts, was a paw withdrawal threshold (PWT), also referred to as 50% paw withdrawal threshold [10,21,24,38,40,41,47,48,49,50,51,54,55,70,71,72,73,74,75,76,77,79,81,82,83,84,86,87,88,89,90,91,92,93,94,95,96,97,98,100,101,104]. Two manuscripts utilized a percent withdrawal threshold normalization of the left to right limbs [37,61]. The final category utilized was the electronic withdrawal measurements, often referred to as the von Frey threshold or threshold value, as seen in 8 manuscripts [15,16,39,67,78,80,85,99].

The PWT measure uses repeated measures with the filaments to calculate the lightest fiber strength in grams with which the mouse withdraws its limb 50% of the time [47,72,101]. This method is also called the up-down method or the Dixon method, which refers to the statistical method used to calculate the 50% withdrawal threshold [32,47,72,101,105]. Many studies employing the 50% withdrawal threshold method used methods similar to those described by Kim et al. and Sun et al. After a five-minute acclimation period for each mouse, Kim et al. applied filaments five times at each strength (starting with the 0.4 g filament), with three-second holding times, and the number of times the animal withdrew its paw was recorded. If the animal withdrew <50% of the time, a heavier filament was used until >50% withdrawal response was recorded. Once the 50% threshold was reached, they stopped increasing strength and recorded the current filament strength as the 50% withdrawal threshold. This test was repeated twice for each animal [47]. The method used by Sun et al. utilized the Dixon up-down method for the calculation of 50% PWT. After habituation to the testing chambers, the fibers starting at the lightest (0.07 g) were applied for 2–3 s. The strength was increased up to the maximum (6 g) until the animal exhibited a response. The next lightest fiber was tested again, and then the next lightest fiber, until no response occurred. They then repeated this up-down method for four more measurements. The results were calculated with the following formula: 50%PWT (g) = 10^Xf+kd^/10,000, where Xf = the value (in log units) of the final von Frey filament, k = tabular value for the response pattern of the last six positive/negative responses, and d = the mean difference (in log units) between von Frey filaments [32,72] (see the manuscript [72], Dixon et al. [105]., and Chaplan et al. [106] for more explanation and the tabular value). The threshold force was calculated twice for each animal and averaged with the sequential measurements, which were separated by at least 10 min [72].

Hwang et al. and Weaver et al. utilized a normalized withdrawal response. Hwang utilized two filament strengths (0.16 g and 0.4 g), while Weaver utilized only the 0.16 g filament. Both habituated the mice to the testing chamber in the two weeks leading up to testing and acclimated them to the chamber for 10 min immediately before testing. The fibers were applied to the plantar surface of the hind paws five times, and the number of withdrawals was recorded. This process was then repeated six times on two different days, equating to 30 measurements. The positive responses on the injured limb (right) were divided by the positive responses on the contralateral limb to obtain normalized values [37,61].

Electronic von Frey tests employ a more straightforward and time-efficient measurement method. In studies using this assay, mice were acclimatized to the testing chamber for 15 to 30 min prior to testing, and the electronic von Frey aesthesiometer was placed on the plantar surface of the injured paw. A gradually increasing force was applied until the peak detector scored the withdrawal of the mouse’s paw [16,78]. Mice were measured in triplicate, and the highest force (grams) was recorded as the withdrawal threshold [16].

While the manual von Frey assay is the most utilized method (featured in 42 manuscripts), it has some disadvantages compared to the electronic von Frey assay. First, it is much more time-consuming and requires a higher level of skill and expertise from users to correctly place and apply the filaments and identify true withdrawal responses [32]. Additionally, the manual method poses a higher risk of acquiring a training effect or response in mice due to the increased number of stimuli (pokes) administered to the plantar surface of the paw. In contrast, the electronic von Frey assay (used in eight manuscripts) reduces the frequency of stimuli administered from upwards of 80 pokes per time point to approximately three per time point: potentially minimizing the risk of such effects [16,32,47,78,81].

For this assay, mice were typically acclimated for 15 to 30 min before measurements [10,16,21,41,84,91]. The mice were then subjected to either manual or electronic von Frey testing. Manual tests used filaments ranging from 0.0008 g (manuscript range 0.0008–0.6 g) [83] to 8 g (manuscript range 0.004–8 g) [40]. No single study used this entire range, and not all manuscripts reported the range of fiber strength. The fiber strength typically started at 0.02–0.07 g for the lightest fibers [41,72,81,92,98,101] and ended at 2–6 g for the heaviest fibers [41,72,75,81,92,98]. Mice typically received multiple rounds of testing to obtain averages, with at least five minutes of resting time between rounds [76,99,101]. Statistical differences between the controls (naïve animals, baselines, or shams) and the DMM-injured animals are typically observed at early time points after injury and last for the duration of the study, with a few exceptions. Most studies (32 studies) utilized baseline measures, and some specifically mentioned blinding (25 studies) (Supplementary Table S1).

Young male mice were found to have differences in mechanical allodynia as early as one day post-injury (PI) [76] to week 30 PI (the farthest time point tested) [101], with most studies falling within this range (Table 1). Some studies did not show significant differences between DMM and controls at some or all of the time points tested. Driscoll et al. only saw significance at week 12 PI and tested weeks 1–12 [70]. Sun et al. saw significance at weeks 3–8 but not at week 2 PI [72]. Geng et al. had significance at weeks 2–8, but not at week 1 PI [79]. Shin et al. saw significance at weeks 4–8 but not at week 2 PI [84]. Miller et al. found significance between DMM and shams at weeks 2–12, but not at week 16 PI [87]. Lin et al. did not find differences between DMM and controls at weeks 1–2 but did at weeks 4–8 PI [88]. Alves et al. had significance at weeks 6, 10–12, but not at weeks 4 and 8 PI [48]. Gowler et al. saw significance at weeks 14–16 but not at weeks 1–11 PI [98]. Kim et al. did not have significance at week 2 PI but did for weeks 5–9 PI [100]. All other studies tested either only one time point or found significant differences between the controls and DMM at all tested time points [24,37,38,41,47,49,61,71,73,74,75,76,77,81,82,83,86,89,90,91,92,94,95,97,101,104].

For the older male mice, electronic von Frey tests found significant differences between controls and DMM animals from weeks two [16,39,85] through 16 PI [67], as the very early and late time points were not tested (Table 1). The only exception or contradiction to those findings was that Li et al. did not observe significance between controls and DMM animals at weeks 1, 2, and 5 PI, but did at weeks 3–4 and 6–8 PI [99]. All other studies tested either only one time point or found significant differences between the controls and DMM at all the tested time points.

Older male mice demonstrated significant differences in mechanical allodynia between the control and DMM groups for von Frey testing from weeks two [39] to 20 PI [50] (Table 1). However, Willcockson et al. found no differences in sensitivity between the contralateral limb and either the baseline or DMM limbs, weeks 4–20 PI [54]. All other studies tested either only one time point or found significant differences between the controls and DMM at all the tested time points [10,21,39,50].

Only 3 studies tested mechanical allodynia in female mice. All the studies tested utilized female mice that were younger than 12 weeks at the time of DMM. Hwang et al. found significant differences between the sham and DMM animals at weeks 2–12 and 16 PI, but not at weeks 14, 18, and 20 [38]. Corciulo et al. used ovariectomized mice and found significantly increased sensitivity to von Frey from the injured to the sham animals at week 2 PI (the only week tested) [40] (Table 1). Malfait et al. did not find any differences from baseline for DMM females at weeks 2–8 PI [55].

Not many studies considered a naïve or non-operated control group and/or ran longitudinal mechanical allodynia tests; however, two that did had interesting trends in the von Frey threshold test [48,101]. As stated above, a risk associated with the von Frey tests, and especially the manual tests, is a potential trained premature withdrawal response if the animal learns that lifting makes the tests end sooner. This risk increases with the duration of exposure to the test or as the number of paw pokes increases. In the studies by Xu et al. and Alves et al., non-significant trends toward increased sensitivity (i.e., decreased 50% PTW values) were observed over time. Although these findings were not statistically significant, and few studies have assessed animals over time in a way that would reveal such trends, they highlight an important consideration for experimental design [32,48,101].

Foot-Brushing and Pin-Prick Tests

A study by Chen et al. utilized two additional mechanical allodynia measures: a custom foot-brushing assay and a pin-prick assay. Similar to the von Frey assay, the mice were accustomed to their environment, and the stimulus was evoked. For the foot brushing assay, the custom brush was used to stroke the bottom of the paw from heel to toe. Mice were scored based on their reaction, from 0–3, with 0 being a fast movement reaction to the stimulus and 3 being multiple episodes of flinching or licking. For the pinprick assay, a small pin was applied to the plantar surface of the paw, without breaking the skin, and repeated 10 times within one minute; the number of times the animal responded was recorded. This study was a short-term study, and it found significant differences between the DMM and baseline animals at days 7–19 PI for both tests [76].

#### 3.1.2. Thermal Sensitivity

Thermal-sensitivity assays are evoked measures of nociception in which a controlled heat or cooling stimulus is applied to a peripheral site (most commonly the paw of the injured limb in the reviewed studies), and the response to the stimulus, such as withdrawal latency, paw lift/licking, or jumping/flinching, is recorded [16,19,32,70]. Common thermal paradigms include hot-plate and radiant-heat methods (e.g., Hargreaves or infrared), cold plate, and chemical cooling (acetone evaporation). The theory behind these assays is that animals with a more painful knee will be able to withstand less heat or cold on the plantar surface of their foot due to the pain in their knee and the referred sensitivity in that limb [32]. As such, these assays are commonly interpreted as measures of referred or secondary sensitization (limb hypersensitivity) and have been used in DMM and other OA studies to capture changes in limb sensitivity associated with the injury [16,19,32,70].

Hot Plate or Hot Allodynia

Hot plate testing is a measure of evoked pain, where researchers measure paws for increased sensitivity to a hot surface (Figure 3). For the hot allodynia tests, animals were typically acclimated to the procedure room before testing [19,107,108]. Some manuscripts performed baseline testing [51,109]. Animals were then placed on a hot plate [14,19,51,73,77,107,109,110,111] or a glass surface for infrared heat testing [24,55,76,82,94,108]. Regardless of the heat source, the outcome measured was latency to withdrawal, where animals either licked, jumped, lifted, or flicked their paw [14,19,24,51,55,73,76,77,82,94,107,109,110,111]. Tests were typically performed in triplicate with a 10-min recovery period. For the temperature-based hot plate tests, the temperatures were set between 50 and 55 °C; animals were tested for a maximum of 60 s, but more typically for 30–45 s to prevent tissue damage [14,19,51,73,77,107,109,110,111]. Studies utilizing direct infrared heat [24,55,76,82] reported a 25-infrared intensity [24,82] with a cut-off time of 10 s to prevent tissue damage [55,76,82]. Studies utilizing an infrared heated glass plate did not report the temperature settings or a maximum time (times reported in Table 2 are based on the maximum value on the y-axis in manuscripts) [94,108].

The results from the manuscripts demonstrate observable significant differences between control animals or baselines and DMM-injured animals for hotplate testing. One study considered female mice (≤12 weeks) (but did not distinguish them from males), and found significant differences between the control and DMM animals at week 6 PI but not at weeks 2, 4, and 8 (Table 1 and Table 2) [55]. One study by Wu et al. examined older male mice and found no significant differences between the controls and DMM animals (Table 1 and Table 2) [111]. Fourteen studies utilized male mice younger than 12 weeks at the time of injury and reported significant differences between the controls and the DMM animals for at least one time point between weeks 2–16 PI. However, not all studies reported these exact findings, as many did not extend to 16 weeks, and many conducted only single-time point testing (Table 1 and Table 2) [14,19,24,51,55,76,77,82,83,94,107,108,109,110]. All but four of the studies showed significant results at all of the time points tested (Table 2).

Cold Plate or Cold Allodynia

Cold plate testing is another method for measuring evoked pain. Researchers measure paws for increased sensitivity to a cold surface (Figure 3). The theory behind this test is similar to the hot plate tests, as they both involve thermal pain responses. One study, using young male mice, tested their sensitivity to cold with a 10 °C plate. All mice withdrew their paws within 20 s. Tests were run in triplicate with a 10-min recovery period. Driscoll et al. found significant differences between the DMM and sham animals only at week 12 PI (Table 1) [70].

Evaporative Cooling/Chemical Sensitivity

Chemical sensitivity was tested using acetone droplets in each of the three relevant manuscripts. Acetone tests are a measure of evoked pain and are utilized as a measure of pain with the principle that the foot of a painful knee will be more sensitive to temperature (or pain) than a healthy limb; as such, increased pain is recorded as a decreased response time to the stimuli. This test is similar to the cold plate testing, as evaporating acetone creates a cooling sensation. In this assay, researchers measure the latency to a response such as licking, flicking, stomping, flinching, or lifting [16,76,109].

In all manuscripts, the procedure for these tests was similar for the young male mice. A drop of acetone (approximately 50 µL) was placed on the plantar surface of the paw. Animals were observed for 30 s [109] to 60 s [16,76] post-application, and the latency to behavioral responses was recorded. Tests were repeated after short breaks, and scores were averaged. Gao et al. saw significant differences between the sham and DMM animals for weeks 2–8 of the study (Table 1) [109]; in contrast, Karuppagounder et al. did not see any differences between the shams and the untreated DMM animals but did see differences at week 8 PI between the treatment groups [16]. Chen et al. conducted a 2-week study and observed no differences between groups [76].

#### 3.1.3. Knee Hyperalgesia

Knee hyperalgesia, also known as pressure application measurement (PAM), is a direct measure of the sensitivity of the knee joint. In this test, the researcher stabilizes the knee, and a force gauge is placed on the medial side of the knee. Pressure is then mechanically increased until the mouse withdraws its knee (Figure 3). A similar test of increasing pressure could be applied to a human until they reach a pain threshold/discomfort [47,49,51,72,87,88,100,114].

Nine manuscripts employed this approach in young male mice, with similar methods. The mouse was securely restrained, and a 5 mm diameter sensor tip was applied to the medial side of the mouse’s knee joint. Pressure was applied at a rate of 30 to 50 g/s for a maximum of 30 s with a maximum force of 450 or 500 g, to prevent joint trauma. The pressure at which the mouse withdrew or displayed discomfort was recorded. Measurements were typically taken in duplicate or triplicate, 10–15 min apart [47,49,51,72,87,88,100,104,114]. Baseline measures were utilized in most studies [47,51,72,87,88,100,104,114]. Differences between the DMM and the sham/naïve/baseline animals were observed as early as 2 weeks PI [72,87,114] and, for most studies, the increased sensitivity persisted until the study conclusion (Table 1) [47,51,72,88,100,104,114]. In the study by Miller et al., the differences between the DMM and the control group spanned weeks 2–12 PI, but at week 16, the sensitivity measures were no longer statistically different [87]. Two studies found significance between the controls and the DMM group starting at week 4 PI [88,104], while another saw significance appear at week 5 [100], another at week 7 [47], and one only tested week 8 [49]. Zaki et al. observed significant differences between the baselines and the DMM and shams from day 3-week 16 PI, but did not observe significant differences between the DMM and shams for any time point (Table 1) [51].

#### 3.1.4. Mechanical Allodynia-Tail

One manuscript looked at the evoked pain response of the animals in the tail by applying the von Frey filaments to the tail. They utilized the same up-down staircase method as the von Frey paw tests, except the filaments were aimed at the mid-ventral tail. Fibers ranging from 0.015 g to 1.3 g were applied to the tail until the fibers bowed and held for 3 s. The fiber with which the mouse exhibited a response (i.e., tail withdrawal) was recorded, and tests were conducted with sequential measures with at least 5 min between assessments. This assay required customization, and baseline measures were performed. No significant differences were found from baseline for either males or females [55].

#### 3.1.5. Heat Sensitivity-Tail

Wu et al. utilized radiant heat to evoke a pain response in older mice of an unknown sex. They gently restrained the mice with a towel, and the mid portion of the tail was heated by focused light. The latency to tail flicking was recorded. At weeks 6–24 PI, the DMM control animals had a significantly different response to the heat than the dietary-treated animals [111].

### 3.2. Spontaneous and Compulsory Measures

In this section, we discuss behavioral tests associated with spontaneous and compulsory measures of pain, behavior, and mobility. Some assays (e.g., cage monitoring) index truly spontaneous, unprovoked behaviors, whereas others (e.g., treadmill walking/running) impose a movement challenge and thus probe how animals respond to an external motor demand [43,69,115,116]. Spontaneous measures capture ongoing, unelicited behaviors that reflect persistent or affective components of pain and disability (mobility, speed, burrowing, nesting, weight-bearing asymmetry, etc.) and have been used specifically to probe ongoing pain states distinct from stimulus-evoked nociception [22,32,68,69,117]. By contrast, compulsory tests require the animal to perform in response to imposed stimuli (e.g., treadmill-based gait platforms); so, they are not “voluntary” by design but still reveal how gait, balance, or locomotor patterns adapt under an external challenge that can unmask pain-related limitations [32,115]. Translationally, spontaneous/compulsory outcomes often align more closely with clinical measures of ongoing OA pain and function [68]. Because many behaviors can be quantified in multiple ways, we group assays by the behavior they measure and explicitly indicate for each whether the readout is spontaneous, compulsory, or both.

#### 3.2.1. Cage Monitoring

Cage monitoring is a putative method used by researchers to measure spontaneous pain/activity in animals. These assays typically take place in the animals’ home cages or other familiar environments, with the monitoring equipment designed to capture spontaneous patterns of behavior and mobility [6,22,74,112,118,119]. The foundation of this approach lies in the idea that animals experiencing pain will exhibit measurable changes in behavior, either compared to their baseline or non-injured controls [6,112,118,119,120]. Similar tests are utilized in humans to measure mobility in clinical trials. Devices like Fitbits or other wearable activity trackers are increasingly used to gather spontaneous activity data in people as a less biased measure of pain and function [121,122]. While humans can verbally report pain, objective metrics from mobility tracking offer additional insights into mobility and behavior that may not be fully captured through self-reporting. These measures enhance the translational relevance of preclinical models and help align animal studies more closely with clinical outcomes in pain and mobility research.

Open field testing encompasses a wide range of methodologies, with varying timeframes, parameters, and analytical approaches. As a result, there is currently a notable lack of consistency across studies, making it challenging to compare behavior/mobility data between studies. Of the reviewed DMM manuscripts, 27 mouse studies [6,10,15,16,21,40,41,48,49,55,61,71,74,75,78,80,81,92,101,111,112,118,119,120,123,124] and one rat [125] manuscript utilized the open-field/activity monitoring type of assay in their studies. Within this, there were 18 different types of monitoring and analysis systems, 13 different groups of parameters, and 15 different test durations (Table A1). Collectively, the open-field/activity monitoring type can be broken down into 4 principal classifications: (i) open-field test (OFT), (ii) wheel running assays, (iii) ethogram-based behavioral analysis, and (iv) digging and burrowing-based behavior monitoring.

The OFT classification of open-field/activity monitoring encompasses all types of OFTs, including overhead cage monitoring and automated activity monitoring. The OFTs were by far the most common type of testing used, with 25 mouse manuscripts utilizing this approach. Within this subtype, 15 studies utilized overhead cage monitoring, and 9 studies utilized cage vibration (Table A1). For overhead cage monitoring, researchers train/familiarize the animals with the testing apparatus, typically in one or two weeks leading up to the baseline measurements [6,61,92]. Animals are then placed in the cage and, after a short acclimation period (allowing the animal to adjust to being moved), the monitoring program begins recording and tracking the animal’s free behavior and movement patterns [6,10,61]. Timeframes of these tests have ranged from three minutes [15,78,80] to 12 h [101] of recording time. Eight studies utilized short-term tests lasting 3–10 min, 5 studies conducted tests in the 15–30 min range, 2 studies used one-hour tests, and 1 study examined results after 12 h. (Table A1). Automated activity monitoring, such as the Laboratory Animal Behavior Observation Registration and Analysis System (LABORAS), is performed in a manner similar to overhead cage monitoring. Instead of using a camera, infrared sensor, or beams to track animal movement, it utilizes calibrated cage vibrations [22,119,123]. Nine studies used automated cage systems to track mobility; the time frames of these tests ranged from 3 [118] to 15 h [41,71,74,75], with 1 study also using a weekly output during lights-on hours [120]. Two studies used the 3–6 h range, and 6 studies used the 14–15 h range (Table A1).

Wheel running, ethogram-based behavioral analysis, and digging and burrowing-based behavior monitoring assays were all used far less frequently than the OFTs. One study utilized the wheel-running assay by leveraging the natural running instinct of mice, placing running wheels in their home cages. Mice were housed individually in this study to facilitate the collection of individual wheel-running data, collecting information on how fast, how far, and how long the animals chose to run [49]. One study also included digging and burrowing behavior, in addition to the OFT, as an ethologically relevant parameter [120].

Despite the differences in collection methods, software/outcomes can analyze similar behavioral patterns, and many different studies do report similar behavioral trends when comparing the DMM injured animals against the control (baseline, shams, and naïve) animals, or even against DMM animals that have been given an experimental treatment. For young male mice (≤12 weeks old), measurable differences were observed between the DMM and other groups from weeks 2 to 30 PI (Table 1 and Table A1). Only one study, which included young female mice, showed statistical differences between the DMM-injured animals and the treated group at week 2 but not at week 8 PI [40]. In contrast, another study showed no differences at any time point [55] (Table 1 and Table A1). Interestingly, the 2 studies using older male mice showed statistical significance between groups at week 8 PI [10,112] (Table 1 and Table A1).

Parameters such as distance traveled, activity or locomotion, mean speed, and rearing were the most reported parameters in the studies (Table A1). These parameters are undoubtedly valuable and provide a broad picture analysis of how the animals are moving; however, except for rearing, they do little to isolate hind limb mobility, which is crucial in understanding the DMM injury. Activities like rearing and top of hut entries isolate the hind limb mobility as the animals have shifted their weight to only the two hind limbs, and any action they perform puts more pressure on the injured limb. As such, any changes from baseline behavior for these parameters are highly relevant. Top-of-hut entries may be particularly insightful, as this indicates the animal’s willingness to propel itself off its hindlimbs [6,27,126]. However, the top-of-hut entries count was an underutilized measure, with only one study examining this factor.

It is tempting to assume that pain in animals always manifests as reduced movement (i.e., decreased distance traveled, mobile episodes, climbing, and mean speed) paired with increased resting behaviors (i.e., increased resting time, time spent in the hut, freezing time, or general immobility). While these patterns can occur, they do not necessarily capture the full spectrum of pain-related responses [22,62,117]. This assumption largely stems from human behavior, where we tend to slow down after injury, recognizing that rest promotes healing and prevents further damage [127,128]. However, this is not necessarily true for other species, particularly prey animals like rodents [32,117].

In the wild, showing signs of weakness can make an animal a target; instead of withdrawing, prey species may overcompensate, becoming hypervigilant or even hyperactive in response to pain [117]. This means that signs of pain may include not only hypoactivity but also hyperactivity, depending on the context. All of this highlights the importance of within-subject comparisons. It can be argued that baseline behavior should always be recorded, and that researchers should assess behavioral changes relative to each animal’s pre-injury activity, rather than relying only on group-level trends or assuming decreased movement always equals pain [6,27,129].

While open field testing already provides valuable data, its full potential is likely limited by the current lack of standardization. The field would benefit from the development of clear guidelines for conducting these tests, including recommendations on duration, frequency, and information on which tests are most likely to help answer specific research questions. Establishing shared best practices would not only improve reproducibility but also enhance the interpretability and translational value of results across laboratories. With greater collaboration and consensus-building in the field, we are well-positioned to unify OFT approaches and strengthen their role in preclinical research.

#### 3.2.2. Gait Analysis

Gait analysis is a valuable and translationally relevant tool for assessing pain, as gait alterations are commonly used in both preclinical models and human clinical evaluations [13,22,23,65]. Gait can be assessed in two main contexts or classifications: (i) spontaneous gait, where animals move at will (e.g., CatWalk™, footprint analysis), and (ii) evoked or compulsory gait response, such as treadmill walking or running. However, the distinction between spontaneous and evoked gait is not always clear-cut. Some argue that all gait assessments reflect an evoked pain response, while others suggest that natural responses to imposed movement can still provide spontaneous behavioral insights [115,116]. For the purposes of this review, we classify gait assessments as either spontaneous (e.g., CatWalk™, footprint tests) or compulsory (e.g., DigiGait™ treadmill systems).

Among the reviewed studies, spontaneous gait analysis was commonly performed using CatWalk™ (11 studies) [34,36,46,55,67,72,79,96,130,131,132], manual inkblot footprint analysis (10 studies) [15,16,48,51,78,80,81,133,134,135], or custom digital systems (2 studies) [60,136]. In these approaches, animals were trained to walk across a surface, and their steps were recorded either digitally or stamped on paper. Digital systems enabled detailed timing parameters, whereas ink-based methods captured foot positioning only (Table A2) [78,96]. Across studies, male mice 12 weeks and younger exhibited significant gait changes following DMM injury, compared to controls or treated animals, from as early as day 1 [130] through to week 16 PI [34,67,132] (Table 1 and Table A2). Older animals (of both sexes) showed spontaneous gait differences between weeks 6–8 PI [136] (Table 1 and Table A2). Prominently, gait changes were often dynamic over time, reflecting shifting compensatory responses. Indeed, significant differences observed at one time point might not persist across other time points, highlighting the value of monitoring multiple gait parameters to better understand the dynamics of gait and the physiological response. For example, Fouasson-Chailloux et al. used CatWalk™ weekly from weeks 0–16 PI and found that, while velocity and duty cycle differences between naïve and DMM animals were significant only at week 1, paw area differences persisted from weeks 1 to 4–16 PI [34]. Contrarily, Shi et al. found differences in right hind/left hind (RH/LH) % print area, % stress, and % swing speed between DMM and sham animals from weeks 2–8 PI, where Fouasson-Chailloux et al. did not observe significant changes in these selected measures (Table A2) [34,96]. This highlights the importance of evaluating multiple gait parameters to accurately capture pain-related functional adaptations and compensations.

Five studies employed compulsory gait analysis using the DigiGait™ treadmill system, at speeds ranging from 17–35 cm/s (walking [137] and running [6], respectively) [6,14,113,137,138]. Similar to spontaneous gait testing (but more extensive), animals used in treadmill analysis required two training periods to become accustomed to walking or running on the apparatus [6]. In the reviewed studies, young male mice showed significant gait differences between the DMM and control/treatment groups from weeks 2 [6] to 12 [14,113] PI. While most studies tested single timepoints, Pezzanite et al. evaluated multiple consecutive weeks and, similarly to the spontaneous gait analyses, found variability in significance across parameters: stance and swing differences at weeks 2 and 4, break stride differences at week 3, and propulsion differences at week 4 [6] (Table A2). This variability underscores the importance of using multiple metrics and incorporating baseline measurements to accurately interpret gait changes. One study also examined older female mice (16 weeks at DMM surgery) and observed significant gait differences at 16 weeks PI for break time, paw area, paw angle, and midline distance between injured and treated animals [138] (Table A2).

#### 3.2.3. Weight Distribution

Weight distribution assays are a method for measuring spontaneous pain in animals. Unlike evoked pain measures, which test a response to stimuli and are generally involuntary, spontaneous measures capture a more natural measure of pain through voluntary compensation, or even subconscious compensation, and can be utilized to measure very early differences in pain pathology [139,140]. Weight distribution or weight-bearing asymmetry (WBA) can be measured either statically or dynamically. For rodents, researchers typically utilize static WBA between the hind limbs (likely because it is much easier and does not require specialized training), with 20 mouse studies employing static WBA of the hind limbs [24,33,35,38,39,50,51,54,67,70,94,98,99,104,118,141,142,143,144,145], and 1 study using a dynamic measure with the front limbs [146]. One group, utilizing rats, developed a dynamic or jump incapacitance test for rats [147] that has the potential to be highly clinically relevant, especially for human athletes. In human studies, WBA is typically measured via dynamic methods, such as sit-to-stand, walking, or stair climbing, which are typically employed over static weight-bearing asymmetry assays [139,140]. The dynamic tests are chosen in humans because they can provide more information from the dynamic measures, testing both functional mobility and a mechanical component [43]. Additionally, it is much easier to ask a human to perform a dynamic task than it is to ask or train a rodent, so the performance bar is set lower [147].

For this assay, mice were acclimated to the testing apparatus on separate occasions prior to the experimental measurements. Static incapacitance (also known as WBA) was assessed using a force plate or electronic weighing scale that incorporated two separate plates. The mice were positioned in a slanted testing chamber, ensuring that their weight was distributed across their hind paws [118] (Figure 3). Once the animals were appropriately situated, weight distribution was recorded over a span of 1 to 5 s, with each measurement being repeated 2 to 3 times. The resulting values were averaged for analysis; some studies reported percentages of weight or WBA, while others normalized the data to baseline values [24,33,35,38,39,50,51,54,67,70,94,98,99,118,141,142,143,144,145]. These different approaches yielded comparable results despite variations in reporting methods or y-axis representations. The only exceptions among the WBA assays were the dynamic measurement conducted by Huesa et al., where animals were monitored for 5 min. In this case, at least 2 min of usable data were required, during which the load on the front paws and the duration spent on them were quantified [146]; and a custom voluntary static incapacitance test by Obeidat et al., where the researchers trained the mice to stand on the platform via a string-pulling task with Cheerio rewards [104].

Young female mice, 12 weeks old or younger at DMM, exhibited differences in WBA between DMM and control animals from week 2 to 20 PI (Table 1 and Table 3) [38]. Notably, only two studies tested this demographic. While Hwang et al. found significant differences for the entire time they tested (weeks 2–20 PI) [38], von Loga et al. only found significant differences later between the DMM and controls at weeks 9–12, and not at the early time points, day 1 through week 8 PI [35].

Three studies evaluated WBA in male mice older than 12 weeks at the time of DMM and reported significant differences between DMM and control groups from as early as week 2 and up to week 20 PI (Table 1 and Table 3) [39,50,54]. In two studies by Wilcockson et al., significant differences were observed through the final time point tested (week 20 PI). One of these studies reported consistent differences between the DMM and sham groups at most weeks, except for weeks 4 and 12 PI [54], while the other found significant differences beginning at week 4 and continuing through the remainder of the study [50].

Seventeen studies examined behavioral differences in male mice that were 12 weeks old or younger at the time of DMM surgery. Among these, significant differences between DMM-injured animals and controls were observed as early as day 1 PI [70,145] and as late as 20 weeks PI (Table 1 and Table 3) [38,143]. Six studies reported consistent and statistically significant differences at all tested time points between DMM and control animals [24,38,51,67,94,142,145]. In contrast, 8 studies reported significant differences at some, but not all, time points [33,35,70,98,99,104,118,143,144]. Individual studies varied in their timelines of significance (Table 3 and Supplementary Table S1).

#### 3.2.4. Balance

Balance is broadly considered an indicator of neurological or cognitive function. Two widely used assays to assess balance in preclinical models are the rotarod and balance beam tests, which evaluate cerebellar function and motor coordination and can detect deficits in motor performance [65,148]. Although balance assessments are rooted in neurological function, they are also influenced by motor impairments [140]. In practice, these tests cannot fully distinguish between neurological and motor dysfunction when animals experience injuries affecting movement. Consequently, both the rotarod and balance beam are commonly used to evaluate balance-related motor deficits or pain-associated impairments [65,148]. In the context of this review, balance is interpreted primarily as a measure of motor dysfunction rather than of neurological or cognitive impairment [148].

Rotarod test

The rotarod test is used to assess mouse mobility as a response to injury or treatment. It primarily assesses balance and motor function, as mice are measured for how long they can stay on a rotating rod and/or how many times they fall off in a given test time (Figure 3).

For this assay, mice were accustomed to this test before measurement. Experimental methods differed slightly across the studies, but the principles and outcome measures were similar. For most studies, researchers placed the mouse on an immobile or slowly rotating rod and allowed it to acclimate before initiating the acceleration. The rods were typically 3 cm in diameter and rotated between 0 and 60 rpm (the most common maximum was 40 rpm), reaching the highest speed at the end of the test time. Tests typically lasted for 5 min (300 s); some studies placed the mouse back on if it fell, while others just recorded the latency to the first fall [38,40,55,81,111,120]. Young female DMM mice showed statistical differences in time spent on the rod compared to sham, baseline, or naive mice at 20 weeks PI. Interestingly, OVX females differed from the intact DMM females at week 2 PI [40] (Table 1). Young male DMM vs. sham/baseline/naive mice did not have differences in rotarod mobility until at least week 8 PI [81]; in other studies, it was later, weeks 10–16 [120], or not until week 20 PI [38] (Table 1). Older males were only tested until week 8 PI, and no significant differences were observed [111].

Balance beam

The balance beam test is used to assess voluntary mobility across a slanted beam or rod. Mice are self-motivated to cross a narrow beam from a bright base area to a dark box at the top (Figure 3). This test primarily assesses balance and motor function, as mice are measured for how quickly they can move across the beam and/or how many times they fall off.

For this assay, mice were acclimated before measurement. Only two studies utilized the balance beam test and included only young male mice. They had similar methods, using light at one end of the beam and a dark box at the other end. Xue et al. utilized a 2 cm diameter meter-long beam/rod angled at 15°, while Qian et al. utilized a 0.9 × 0.9 × 20 cm beam, with the box 40 cm off the ground. Both recorded the time to walk across the beam. Xue et al. also recorded the falling frequency [81,133]. Significant differences for young male mice were observed as early as week 4 PI and through week 12 in one study [133] and weeks 8–12 in the other (Table 1) [81].

#### 3.2.5. Hang Test

This test assesses the ability of animals to effectively utilize all four limbs, which is crucial for their activities and survival, particularly since lab mice often climb to access food. The primary focus of this assessment is to measure spontaneous pain. In this experiment, researchers positioned mice upside down on a wire mesh and timed how long they could hang [100,109]. The timer was stopped either when the injured limb’s paw lost contact with the mesh, or when the mouse fell off the mesh, or reached max time 1 [100] or 3 [109] minutes. Two studies assessed the inverted hang time in young male mice. Gao et al. found that at all timepoints they tested (day 3–week 8 PI), there were statistical differences between the DMM and Sham groups [109]. Kim et al. tested at week 8 and found that the time point was statistically different between the DMM and sham groups (Table 1) [100].

#### 3.2.6. Grip Strength

Wu et al. utilized a compulsory mouse grip strength test with a mouse grip-strength meter. They used this assay as a musculoskeletal function test and measured both fore- and hind-limb strength. They found differences in fore-limb strength between their dietary treatment groups and the control diet at weeks 6 and 24 PI, but not at week 16, and no differences between groups were found in the hind-limb grip strength (which would be more closely related to the injury/PTOA) [111].

#### 3.2.7. Mouse Grimace Scale

Also known as the composite pain score of facial expression, this approach looks at a mouse’s facial expressions to assess overall pain. Mice were placed in an observation cage for 1 min to acclimate to the cage, and then they were recorded for 3 min [36]. The facial expressions (orbital tightening and ear position) and body conditions (spontaneous behavior, posture, coat condition, eye condition, body condition, wounds present, and movement) were scored from 0–11 from a table by Jirkof et al. [149]. Baseline measures, acclimatization, and accustomization were utilized for this measure. Rapp et al. evaluated young male mice and found that grimace scores were only significantly different between the sham and DMM animals at week 11 PI. It is important to note that other tests also found differences in behavior between these groups at week 12 PI (Table 1) [36].

#### 3.2.8. Nest Complexity

The complexity of the nest in the mouse is a spontaneous behavioral indicator of group wellbeing and self-care, where more complex nests indicate improved wellbeing (Figure 3). Rapp et al. utilized nest complexity of mice given more natural nesting materials and scored them from a scale of 1–5 based on the system developed by Hess et al. [36,150]. Rapp et al. evaluated young male mice and found that the nests were only significantly different between the sham and DMM animals at week 11 PI (Table 1) [36].

### 3.3. Other

In this section, we have included two other assessments related to OA that were present in one or more studies.

#### 3.3.1. Knee Edema

Three studies measured the diameter of the mouse knee joints at the final time point to assess knee edema. Knee edema is not a direct measure of nociception, but because joint swelling is commonly associated with pain and functional limitation, measures of swelling or related inflammatory/structural outcomes were considered clinically relevant endpoints for this review. All three studies utilized young male mice and only examined week 12 PI, when they observed significant differences in the diameters of the DMM and sham knees (Table 1) [15,78,80].

#### 3.3.2. Microglia Assessment

A study by Tran et al. examined active microglia in the central nervous system as indicators of pain [90]. Microglia cell activation contributes to synaptic plasticity transmission associated with chronic pain [90,151]. Spinal cord sections were collected from mice and stained with an immunoreactive microglial marker (ILBa1-ir). A ratio of the process length to the soma diameter was used to classify the microglia as resting (>double length to diameter) or activated (<double length to diameter). The number of activated microglia was counted at weeks 4, 8, and 16 PI. At weeks 8 and 16 PI, the DMM animals had significantly more activated microglia than the naïve or sham animals (Table 1) [90].

## 4. Discussion–Rats

### 4.1. Evoked Measures

#### 4.1.1. Mechanical Allodynia

Mechanical allodynia testing for rats was very similar to that of the mice. Five assays utilized mechanical allodynia tests, with four assays using von Frey [17,56,152,153] (two assays electric [56,152]), and one unknown mechanical allodynia test [154]. The filament strengths for the manual von Frey tests were not specified in either of the manuscripts. Male rats less than 12 weeks old at the time of DMM were the only rats tested for mechanical allodynia. The DMM animals exhibited significantly increased mechanical allodynia compared to the controls (baseline, naïve, and shams) at weeks 1–12 PI (Table 4) [152]. Week 12 was the latest time point tested in these animals. Zhang et al. only tested week 2 PI [56], while Deng et al. tested weeks 2–12 PI [17]; both groups saw significant differences between the DMM and controls. None of the studies showed any contradictions to results from other studies for the timeline of increased sensitivity to von Frey [17,56,152,153,154].

#### 4.1.2. Thermal Sensitivity

Hot plate or hot allodynia

The methodology for the rats was very similar to that of mice. One study utilized a hot plate set at 50 °C [56], another study used direct infrared heat [17], and another study used an unspecified thermal test [154]. All 3 studies tested young male rats, and only Zhang et al. demonstrated significant differences between the controls and DMM animals at week 2 PI (Table 4) [56].

Cold Plate or Cold Allodynia

One study utilized cold plate testing in rats at a temperature of 0 °C. No max time was stated, but all rats withdrew their paws within 150 s. Tests were run in triplicate with a 10-min rest period. Zhang et al. demonstrated significant differences between the controls and DMM animals at week 2 PI (Table 4) [56].

### 4.2. Spontaneous and Compulsory Measures

#### 4.2.1. Cage Monitoring

One study with young male rats employed a unique cage monitoring assay. For this assay, rats lived in a multi-level rat colony with location tracking. Rats were chipped and trained to jump to different levels, and when they jumped, a computer recorded the cage movement. With this assay, the researchers isolated a highly relevant movement parameter to measure, as it assesses the animals’ willingness to jump off an injured limb. These DMM-injured animals had statistically different measures from controls only at week 1 PI [125] (Table A1 and Table 4).

#### 4.2.2. Gait Analysis

Six studies employed gait analysis in rats, with methodologies largely mirroring those used in mice, apart from adjustments for body size and the addition of a maximum-speed treadmill running test [20,57,125,147,153,155]. For instance, treadmill walking speed increased from 17–18 cm/s in mice [113,137] to 25 cm/s in rats [57] to account for size-related gait differences. Four studies utilized spontaneous gait measures [125,147,153,155] and two used compulsory treadmill-based analysis [20,57] (Table 5).

Among the spontaneous gait studies, three focused on young male rats and observed significant differences between the DMM-injured and control or treated groups at weeks 8 and 19 PI. The intervening time points showed no significant changes. Notably, the study extending to 19 weeks PI assessed only one parameter (% print length of the contralateral limb), while the other two studies measured gait only at week 8 PI (Table 4 and Table 5) [125,153,155]. One study using older female rats assessed spontaneous gait weekly through week 14 PI but found a significant difference in % print length of the contralateral limb only at week 1 PI (Table 4 and Table 5) [147].

The two studies using compulsory treadmill running assessed young male rats and reported significant gait differences between DMM and control or treated animals from weeks 1–6 PI. However, not all gait parameters showed significant changes throughout this period (Table 4 and Table 5) [20,57].

#### 4.2.3. Weight Distribution

WBA for rats was assessed using methods similar to those for mice. Animals were habituated to the testing room and chamber, placed in the chamber with one paw on each force plate, and measured for three seconds. Tests were measured in triplicate and averaged [33]. Gowler et al. utilized male rats younger than 12 weeks at injury and tested weight-bearing asymmetry weekly from week 0–16 PI. They did see differences between the injured and sham animals from week 6–16 PI. It is also important to note that Gowler et al. utilized a less aggressive form of the DMM injury than traditional for these rats [33]. Westhof et al. developed a dynamic weight-bearing assay, in which female rats older than 12 weeks old were trained to jump voluntarily from a force plate, and the computer recorded the difference in distribution during the jump (see the manuscript for more details). They tested weeks 1–14 PI and saw significant differences between the DMM and control animals at all tested timepoints (Table 4) [147].

#### 4.2.4. Balance

Rotarod

Similar to the mouse test, rats were placed on a 60 mm diameter rod that was slowly accelerated from 5–40 rpm [16,58] (or max 30 rpm [156]) over 30 s. The time to first fall and the number of falls were recorded. Young male rats had statistically different ride times at weeks 2–4 PI (Table 4) [58]. Older female rats had statistical differences observed for weeks 1–7 PI (Table 4) [156].

### 4.3. Other

#### Knee Edema

A study by Liu et al. measured the circumference of the knees of rats post-surgery for 4 weeks. They found that the young male DMM rats had significantly increased joint circumference or edema compared to sham animals, starting at week 2 PI and continuing through to the study conclusion (Table 4) [57]. Westhof et al. measured the knee diameter of 14-week-old female rats using calipers weekly from 1–13 weeks PI and saw significant differences in the DMM and sham animals every week (Table 4).

## 5. Consideration of Analgesics

The scope of this review focuses primarily on differences in animal behavior and mobility between control animals and DMM-injured, untreated animals, and if/when these changes are seen between these groups for the different assays. However, as pain is the presumed component causing these changes, it is important to take a brief moment to address how pain treatments affect animal behavior and mobility. As this topic is worthy of its own review, we will only briefly discuss how treatments with painkillers/analgesics compare to injured, untreated animals and controls. We have highlighted 13 of the reviewed studies that used more traditional analgesics, such as opioids that act directly, and more indirect routes such as NSAIDs, steroids, traditional eastern medicines, and anti-inflammatory agents. It is important to note that many of the reviewed studies tested the effects of therapeutics to reduce pain and inflammation and should be considered in a larger review of this topic; however, this is not the topic of this review. For a more in-depth analysis, please see Supplementary Table S1.

Briefly, for studies that considered cage/activity monitoring:

- Wan et al. treated mice with baicalein or baicalein + ferroptosis inhibitor ferrosatin-1 (fer-1), and treatment with baicalein significantly helped animals maintain mobility in comparison to the DMM group at all measured weeks (2, 4, and 10), but treatment with baicalein and fer-1 significantly improved mobility from baicalein alone. Treated animals were closer to uninjured control mobility for both measures [75].
- Sun et al. treated mice with celecoxib. At 8 weeks PI, for the celecoxib-treated animals, there were significant increases in distance traveled, mean speed, and active time compared to the vehicle controls. The treated group was also equivalent to the sham [49].
- Tang et al. treated mice with 4-octyl itaconate. Starting at week 4, treatment with 4-OI ameliorated behavior/mobility changes seen in the DMM animals, equivalent to shams for both distance traveled and average speed [92].
- Xu et al. treated mice with recombinant human midkine (rhMK) and saw that treatment increased rearing and movement to levels similar to controls. However, at week 8 PI, the rhMK-treated group decreased temporarily to the level of the DMM vehicle group [101].

Briefly, for studies that considered gait analysis:

- Liu et al. treated mice with betulinic acid (BA). At 8 weeks PI, the high-dose injection of BA significantly improved gait for base of support (BOS), right hind paw stride length, and right-left hind paw distance compared to the vehicle controls. Low dose of BA treatment saw improvements from the vehicle for BOS. Improved measures were similar to the shams [134].
- Wang et al. treated mice with Eucommia ulmoides Oliv. and Glycyrrhiza uralensis Fisch. (E.G.). At week 12 PI, the DMM animals had significantly altered gait compared to the shams. Treatment with E.G. ameliorated the effects of the DMM injury on the gait [14].
- Xu et al. treated mice with Glycyrrhiza uralensis Fisch. (GC). At week 12 PI, the DMM animals had significantly altered gait, and treatment with GC helped animals maintain a gait similar to the shams [113].
- Westhof et al. rats treated with Zilretta. They measured only the percent print length of the contralateral limb for gait and saw differences between the control and DMM groups only at week 1 PI, with all other weeks measured displaying no differences in gait for any of the three groups. The treatment with Zilretta mirrored the vehicle treatment for gait [147].

Briefly, for studies that considered weight distribution analysis:

- Jin et al. treated mice with Prostaglandin E receptor 4 (EP4), grapiprant, HL-43, and celecoxib. They saw that the EP4-inhibited mice had a more equal distribution than untreated DMM animals at 8 weeks PI [24].
- Hwang et al. treated mice with capsazepine (CPZ) and saw at 8 and 10 PI that the male mice had a significant improvement in weight-bearing symmetry compared to untreated animals when given CPZ. Females did not exhibit different pain behaviors when treated [38].
- Westhof et al. saw that at 1 week PI, the DMM rats had significantly decreased percent incapacitance of the contralateral limb compared to controls, and that at 2 and 5 weeks PI, the Zilretta treatment significantly increased symmetry [147].

Briefly, for studies that considered mechanical allodynia:

- Wan et al. saw that in WT mice, both treatments with baicalein and baicalein + fer-1 significantly reduced sensitivity in comparison to DMM for every tested week, and that in KO mice, only the baicalein + fer-1 significantly reduced sensitivity in comparison to DMM for every tested week [75].
- Jin et al. saw that the EP4 KO mice had decreased sensitivity in comparison to the vehicle controls at 8 weeks PI. All 3 drug treatments in the WT mice demonstrated significantly reduced sensitivity in comparison with the untreated control at week 6 PI [24].
- Sun et al. saw that at 8 weeks PI, celecoxib-treated mice had significantly reduced sensitivity compared to the vehicle controls [49].
- Miller et al. treated mice with morphine or clozapine-N-oxide (CNO). At 12 weeks, PI treatment with morphine significantly reduced sensitivity for WT mice compared to vehicle. Treatment with CNO significantly reduced sensitivity to von Frey at 8 weeks PI in Nav mice 1–2 h after treatment, but not 4 h. Treatment at 4, 8, and 16 weeks PI showed no benefit to CNO therapy [87].
- Tang et al. saw that the treatments with 4-OI ameliorated the effects of the DMM injury in mice [92].
- Xu et al. saw that treatments with rhMK reduced sensitivity compared to the DMM-injured mice starting at 2 weeks post-treatment (week 20 PI) in both the injured and contralateral limbs [101].
- Hwang et al. saw that the treatment with CPZ did not decrease sensitivity to the von Frey filaments for male or female mice [38].

Briefly, for studies that looked at thermal sensitivity to heat:

- Jin et al. found that all 3 treatments led to decreased sensitivity to the thermal hyperalgesia tests at week 6 PI when compared to untreated mice [24].
- Liu et al. treated mice with U50,488H and found that the treatment reduced sensitivity to heat at 8 weeks PI in comparison to the untreated mice [110].
- Wang et al. found that at week 12 PI, DMM mice had significantly increased sensitivity to heat. Treatment with E.G. ameliorated this [14].
- Xu et al. saw that at week 12 PI, DMM mice had significantly increased sensitivity to heat compared to shams and that GC treatment significantly decreased the sensitivity seen in DMM animals [113].
- Gao et al. treated mice with Tetrandrine (Tet), celecoxib (CXB), or indomethacin (INDO) and found that mice treated with any of the treatments had significantly decreased sensitivity weeks 2–8 PI [109].

Briefly, studies that considered chemical sensitivity (acetone) tests:

- Gao et al. saw that all three treatments significantly decreased sensitivity to acetone cooling compared to untreated mice at weeks 2–8 PI [109].

Briefly, studies that considered knee hyperalgesia:

- Sun et al. found that at 8 weeks PI, celecoxib-treated mice had significantly reduced sensitivity compared to the vehicle controls [49].
- Miller et al. saw that treatment with morphine significantly reduced knee sensitivity 15 to 75 min after administration at week 4 in WT mice and that Nav mice had a significant reduction in sensitivity up to 4 h after CNO therapy compared to the controls at week 4 PI [87].

Briefly, studies considering inverted hang tests:

- Gao et al. saw that all three treatments significantly increased hang time from the vehicle control for the inverted wire mesh hang test at week 4 PI [109].

## 6. Conclusions

While many studies using the DMM model have successfully demonstrated differences in evoked pain responses (e.g., mechanical or thermal sensitivity), increasing evidence suggests that spontaneous measures (e.g., gait, weight distribution, and open field mobility) may more accurately capture OA-related pain. Different pain modalities activate distinct neural pathways, and studies in both humans and animals have shown that evoked and spontaneous pain are not always correlated [43,50,68]. For instance, Parks et al. found that patients with knee OA did not differ from healthy controls in evoked pain sensitivity (specifically, peak pain rating); however, they showed clear differences in spontaneous pain, aligning more closely with chronic pain conditions, such as back pain [68]. Similar limitations likely apply in animal models, raising concerns about the sensitivity and ecological validity of evoked tests, such as the von Frey assay, which measures referred rather than localized joint pain [43].

Although most studies reviewed here found significant group differences using von Frey testing, it is essential to acknowledge potential pitfalls. Animals may exhibit learned behaviors over time, such as withdrawing their paws earlier to reduce exposure, especially with repeated testing [32]. While this was not consistently reported across studies, some trends of increasing sensitivity in naïve animals were observed [48,101]. To mitigate these effects, researchers should minimize stimulus exposure time, utilize automated systems such as electronic von Frey devices, and ensure thorough habituation to the testing environment. Including baseline measurements is also critical, as it enables within-subject comparisons, enhances statistical power, and controls for individual variability.

Spontaneous pain assays offer important advantages in this regard. Tests that rely on voluntary behavior, such as gait analysis, open-field testing, and weight-bearing assays, can reveal pain-related changes that more accurately reflect human OA [43]. Our review found robust evidence that these measures distinguish between DMM-injured and control animals, and in many cases, between the treated and untreated groups. This supports their utility not only in assessing disease progression but also in evaluating therapeutic efficacy.

However, several areas of the field require further standardization. Among the reviewed studies, protocols for weight distribution and rotarod testing were relatively consistent, improving cross-study comparability. In contrast, mechanical allodynia protocols varied widely. We strongly recommend the adoption of electronic von Frey systems to enhance precision, reduce human bias, and minimize potential training effects.

Complex behavioral assays, such as gait and open field testing, would also benefit from standardization in both parameter selection and testing duration. Studies used testing times ranging from 3 min to 17 h, complicating comparisons. Additionally, studies considering using open-field testing should opt for automated tracking software to increase objectivity and reduce the need for labor-intensive analysis. Similarly, gait analysis lacks consensus on which parameters are relevant, and there is incredible variability between gait collection methods. Future studies may want to prioritize automated systems, such as CatWalk™ and DigiGait™, as they provide rich spatiotemporal data and are preferable to manual methods, such as inkblot footprint analysis, which only provide spatial data. Future research should address issues related to timeframes for data collection, the speed at which compulsory gait data is collected, and which parameters are relevant to establish clear guidelines for different research aims. Additionally, it should address these concerns differently for males and females, given the differences in disease progression for OA, in the hope of increasing the translatability to human studies and treatments.

Finally, baseline behavioral measurements and blinding should be standard practice. Baseline data allow researchers to assess individual changes over time, reducing inter-animal variability and increasing the reproducibility and rigor of pain and mobility research in DMM models [117]. Blinding was not a factor considered in many of the studies, and traditionally has been considered more important for human-scored assays, such as histology, but it is an important factor that, when not included, can introduce biases. We strongly urge future behavioral, mobility, and pain studies to blind the researchers to treatment groups for data collection and analysis. This is especially important if the researchers are applying any stimuli directly to the animal and/or watching for responses [22,32].

In summary, this review highlights the increasing importance of behavioral and pain assessments in OA research, utilizing the DMM model, especially given the recent findings that pain responses are likely a larger driving factor for OA severity than histology or radiology scores [42]. As functional mobility becomes a central focus in preclinical OA studies, careful consideration of behavioral testing design is essential. This review provides a comprehensive overview of the assays employed to date, indicating when specific behavioral or pain-related changes typically emerge, and identifies areas that require greater standardization. By improving methodological consistency, prioritizing spontaneous behavior assays, incorporating baseline measurements, blinding, and aligning with the principles of replacement, reduction, and refinement (the 3Rs), the field can significantly enhance the translational relevance of animal studies and accelerate the development of more effective OA treatments.

### Recommendations

Findings from this review reinforce the importance of thoughtful experimental design in behavioral and pain assessment following DMM-induced osteoarthritis. Across all assays, proper animal acclimation remains essential. Acclimation sessions should match the testing conditions in terms of time, space, and personnel, and be equal to or longer than the planned test duration. Likewise, baseline behavioral measurements—ideally collected in duplicate—are critical for reducing inter-animal variability and enabling meaningful within-subject comparisons.

While a range of behavioral tools are in use, increasing evidence supports a shift toward spontaneous behavior assessments, such as open-field testing, over traditional evoked pain measures. When evoked tests are used, researchers should opt for modern technologies (e.g., electronic von Frey) that reduce testing time, minimize stress, and limit learned avoidance behaviors. Similarly, newer platforms for gait and activity monitoring—such as CatWalk™, DigiGait™, and automated open-field systems—are recommended due to their ability to capture multiple parameters and better reflect functional impairment.

Emerging tools, such as dynamic weight-bearing and jump incapacitance, offer promising translational relevance and would be valuable additions to mouse-based OA research. Finally, studies should consider sex-specific responses, as biological differences can impact both behavioral and pathological outcomes. These strategies will support greater reproducibility, improve cross-study comparisons, and increase the translational value of rodent PTOA models.

## Figures and Tables

**Table 1:** Onset and (If Applicable) Duration of Statistically Different Behavior Post-Injury Between DMM and Control Mice.

Test	Males ≤ 12 Weeks	Females ≤ 12 Weeks	Males > 12 Weeks	Females > 12 Weeks
Mechanical Allodynia	Day 1–Week 30	Weeks 2–12 and 16	Weeks 2–20	
Hot Plate	Weeks 2–16	Week 6		
Cold Plate	Week 12			
Chemical Cooling	Weeks 2–8			
Knee Hyperalgesia	Weeks 2–16			
Mechanical Allodynia-Tail			Week 8	
Heat Sensitivity-Tail				
Cage Monitoring	Weeks 2–30	Week 2	Week 8	
Spontaneous Gait	Weeks 1–16		Weeks 6–8	Weeks 6–8
Compulsory Gait	Weeks 2–12		Week 16	Week 16
Weight Distribution	Day 1–Week 20	Weeks 2–20	Weeks 4–20	
Rotarod	Weeks 8–20	Week 20		
Balance Beam	Weeks 4–12			
Grimace and Nesting	Week 11			
Hang Test	Day 3–Week 9			
Grip Strength			Week 8	
Knee Edema	Week 12			
Microglia Assessment	Weeks 8–16			

**Table 2:** Outcomes of Hot Allodynia Testing Indicating Statistical Differences in Weeks Post-Injury Between DMM and Control Mice.

Test Type	Temperature	Test Duration	Males ≤ 12 Weeks	Females ≤ 12 Weeks	Males > 12 Weeks	Ref
Radiant heat	20% active intensity	<30 s	* 6 ns 2, 4, 8 weeks	* 6 ns 2, 4, 8 weeks		[55]
Hotplate	52 ± 1 °C	<20 s			ns week 8	[111]
Radiant heat	40 W	20 s cut-off	* week 8			[108]
Infrared	25 infrared intensity	<20 s	* week 6			[24]
Hotplate	NA	<25 s	* 6, 8 weeks			[83]
Hotplate	55 °C	45 s cut-off	* 5, 9, 13 weeks			[19]
Hotplate	55 °C	45 s cut-off	* 4, 6, 8, 10, 12 ns week 2			[107]
Hotplate	55 °C	45 s cut-off	* week 8			[110]
Hotplate	NA	<30 s	* 2, 3, 4, 5, 6, 7, 8 weeks			[94]
Hotplate	55 °C	30 s cut-off	* 8, 12, 16 ns 2, 4 weeks			[112]
Hotplate	52 °C	60 s cut-off	* 4, 8, 12, 16 ns 1, 2 weeks			[51]
Hotplate	50 ± 0.1 °C	<30 s	* week 12			[14]
Hotplate	50 °C	<30 s	* week 12			[113]
Infrared	25 infrared intensity	<20 s	* week 8			[82]
Infrared	25 infrared intensity	<20 s	* week 8			[76]
Hotplate	54 ± 0.1 °C	30 s cut-off	* 2, 4, 6, 8 weeks			[109]

* Indicates statistical significance at the given weeks; ns signifies non-significance at the given weeks.

**Table 3:** Outcomes of Weightbearing Asymmetry, Indicating Statistical Differences in Weeks Post-Injury Between DMM and Control Mice.

Males ≤ 12 Weeks	Females ≤ 12 Weeks	Males > 12 Weeks	Ref.
* 11, 12 weeks ns day 1–3, weeks 2–10			[70]
* 1–3 days, 16 weeks			[145]
* 8, 10–12 weeks ns days 1–3, weeks 1–7, 9	* 9–12 weeks ns days 1–3, weeks 1–8		[35]
* 12, 14, 16 ns days 3, 7, 10 ns weeks 2, 4, 6, 8, 10			[118]
* week 8 (between treatments, no controls shown)			[24]
		* 4, 8, 12, 16, 20 weeks	[50]
		* 4, 6, 8, 10 ns week 2	[39]
		* 8, 16, 20 ns 4, 12 weeks	[54]
* 13–16 ns 1–12 weeks			[33]
* 8, 10, 12, 14, 16			[67]
* 2, 4, 6, 8, 10, 12, 14, 16, 18, 20 weeks	*2, 4, 6, 8, 10, 12, 16 ns 14, 18, 20 weeks		[38]
Stats NA (see Supplemental Table S1)			[146]
* 6–8 ns 2–5 weeks			[94]
* week 10–12 (between treatments, no stats to baseline)			[144]
* day 3, weeks 1, 2, 4, 8, 12, 16			[51]
* 14–16 ns 1–13 weeks			[98]
* 2, 5–8 ns 1, 3–4 weeks			[99]
* 11–20 ns day 1, 3, weeks 1–10			[143]
* week 8			[142]
ns			[141]

* Indicates statistical significance at the given weeks; ns signifies non-significance at the given weeks.

**Table 4:** Onset and Duration of Statistically Different Behavior Post-Injury Between DMM and Control Rats.

Test	Males ≤ 12 Weeks	Females ≤ 12 Weeks	Females > 12 Weeks
Mechanical Allodynia	Weeks 1–12		
Hot Plate	Week 2		
Cold Plate	Week 2		
Cage Monitoring	Week 1		
Spontaneous Gait	Weeks 8–19	Week 1	
Compulsory Gait	Weeks 1–6		
Weight Distribution	Weeks 6–16		Weeks 1–14
Rotarod	Week 2		Weeks 1–7
Knee Edema	Week 2		Weeks 1–13

**Table 5:** Gait Analysis Results—Rats.

Test Style	Parameters Shown	DMM Compared Against	BASELINE	Timepoints Shown	Stance	Paw Area	Stride/Stance/Step Length	Stride/Stance/Step Width/BOS	Velocity	Sex and Age at Time of Injury	Ref.
Catwalk	% print length of contralateral limb	healthy	yes	weeks 0, 2, 4, 6, 8, 10, 19		* 19 ns 2–12				males 8–9 weeks	[125]
Footprint analysis	stride length, print area	sham and treated	no	week 8		* 8		* 8		males 8 weeks	[153]
Footprint analysis	base of support (BOS), stride length, and print area	sham and treated	no	week 8		* 8	* 8	* 8		males 12 weeks	[155]
Compulsory treadmill running	maximum running speed	sham and treated	yes	weeks 0, 2, 4, 6					* 6 2–4	males 10 weeks	[20]
Compulsory treadmill running (DigiGait) 25 cm/s	stride length, paw area, paw weight, pose duration	sham and treated	no	weeks 1, 2, 3, 4	pose duration * 1–4	area * 1–4 weight * 1–4		* 1–4		males 8 weeks	[57]
Catwalk	% print length of contralateral limb	naïve and treated	yes	weeks −1, and 1–14 every week		* 1 ns 2–14				Females 13–14 weeks	[147]

* Indicates statistical significance at the given weeks; ns signifies non-significance at the given weeks.

## Data Availability

No new data were created or analyzed in this study. Data sharing is not applicable to this article.

## Supplementary Materials

The following supporting information can be downloaded at https://www.mdpi.com/article/10.3390/biomedicines13122886/s1. Table S1: Assays, results, and summaries of reviewed articles.

## Abbreviations

The following abbreviations are used in this manuscript:

OA	Osteoarthritis
PTOA	Pots-traumatic osteoarthritis
DMM	Destabilization of the medial meniscus
ACLT	Anterior cruciate ligament transection
PI	Post-injury
PWT	Paw withdrawal threshold
OFT	Open-field test
LABORAS	Laboratory Animal Behavior Observation Registration and Analysis System
WBA	Weight-bearing asymmetry
OVX	Ovariectomized

## Appendix A

**Table A1:** Cage Monitoring/Open Field/Mobility Test Results.

Activity Monitoring Type	Test Duration	Parameters Shown	DMM Compared Against	Baseline Measure	Timepoints Measures Shown	Distance Traveled/Track Length	Walking/Ambulation/Locomotion/Activity	Mean Speed/Velocity	Cimbing	Rearing	Grooming	Immobile/Inactive/Rest	Mobile Episodes	Time in Hut	Top of Hut Entries	Dig/Burrow	RDI	Sex, Age at Injury	Ref
LABORAS (Assessment of Spontaneous movement)	15 h	Assessed locomotive behaviors (distance traveled, climbing, and rearing) overnight	sham and/or naïve	no	weeks 4, 8, 16	* 8, 16 ns 4			* 8, * 16 ns 4	* 8, * 16 ns 4								males 10 weeks	[71]
LABORAS (Assessment of Spontaneous movement)	3 h	grooming, activity, climbing, immobility, measured viasingle cage vibration	sham	no	week 8		* 8		* 8		ns 8	* 8						males 10–12 weeks	[118]
LABORAS (assessment of spontaneous movement)	15 h	travel distance, average walking speed, rearing frequency, and rearing duration	control and treatment	no	weeks 2, 6, 10	* 6 ns 2, 10		ns 2–10		frequency * 6, ns 2, 10 duration * 6, ns 2, 10								males 10 weeks	[74]
LABORAS (Assessment of Spontaneous movement)	14 h	grooming, locomotion, climbing, immobility via single cage vibration	baselines, treatments, and naïve	yes	weeks 0, 4, 8, 12, 16	* 4–8 ns 12–16	* 4 ns 8–16		* 4–8 ns 12–16									males 10–12 weeks	[123]
LABORAS (Assessment of Spontaneous movement)	14 h	climbing, locomotion, eating, drinking, immobile, and grooming	baselines, treatments, and naïve	yes	weeks 0, 4, 8, 12, 16		* 8–16 ns 4		* 4–8 ns 12–16	ns	ns	* 8–16 ns 4						males 10 weeks	[119]
LABORAS (Assessment of Spontaneous movement)	15 h	distance of locomotion, average speed of locomotion, rearing frequency, and rearing duration via single cage vibration	baselines, shams, and treatments	yes	week 0, 2, 6, 10	* 2–10		* 2–10		frequency * 2–10 duration * 2–10								males 12 weeks	[41]
LABORAS (assessment of spontaneous movement)	15 h	average speed, distance traveled	control and treatments	no	weeks 2, 6, 10	* 2–10		* 2–10										males 10 weeks	[75]
Open field analysis (Zhenghua Technology)	3 min	relative activity, active time, distance, mean speed	shams, controls, and treatments	no	week 12	* 12	relative activity * 12 active time * 12	* 12										males 12 weeks	[78]
Open field analysis (Zhenghua Technology)	3 min	relative activity, active time, distance, mean speed	shams and treatments	no	week 12	* 12	relative activity * 12 active time * 12	* 12										males 12 weeks	[80]
Open field analysis (Zhenghua Technology)	3 min	relative activity, active time, distance, mean speed	shams and treatments	no	week 12	* 12	relative activity * 12 active time * 12	* 12										males 12 weeks	[15]
Open field analysis (Activity Monitor: Med Associates)	30 min	distance traveled, ambulatory time, ambulatory counts, ambulatory episodes, resting time, average velocity	shams and treatments	no	week 8	ns 8	ns 8	ns 8				ns 8						males 12 weeks	[16]
Open field testing (EthoVision XT 11)	30 min	distance, average speed	shams and treatments	yes	weeks 0, 4, 8, 12, 16	* 4–12		* 4–12										males 9 weeks	[92]
Open field testing (Smart Video Tracking Software (Panlab))	10 min	total distance traveled, mean velocity (excluding the immobility time), and immobility time.	naïve and shams	no	weeks 4, 6, 8, 10, 12	ns 4–12		* 10 ns 4–8, 12				ns 4–12						males 12 weeks	[48]
Open field testing (SMART, Panlab SL, Spain)	6 min	distance traveled, rearing count	treatments	yes	week 0, 4, 8, 12	* 12 ns 4–8				* 4, 12 ns 8								males 10 weeks	[124]
Open field testing infrared array sensors (Taimeng, Chengdu, China)	12 h	mobile episodes, rearing	naïve, shams, and treatments	yes	weeks 14, 16, 18, 20, 22, 24, 28, 30					* 14–30			* 14–30					males 8 weeks	[101]
Open field analysis( VersaMax activity monitors)	20 min	Distance traveled, ambulatory movements, resting time	treatments	yes	weeks 0, 6, 12	* 12 ns 6	* 12 ns 6					* 12 ns 6						males 12 weeks	[61]
multi-rodent tracking system (open field)	1 h	total distance of movement	shams and treatments	no	weeks 8, 12	* 8–12												males 12 weeks	[81]
Voluntary Wheel Running	24 h	active time, distance traveled, and mean speed	shams and treatments	no	week 8	* 8	* 8	* 8										males 12 weeks	[49]
Observations of digging and burrowing behaviors Digital Ventilated Cages (DVCs) Weekly regulatory disruption index (RDI)	unknown weekly (lights on period)	digging duration, # burrows, RDI	baselines, shams, and treatments	yes	weeks -1 through 16 (weekly)											Digging * 16 ns 1–15 burrowing * 16 ns 1–15	* 16 ns 1–15	males 10–12 weeks	[120]
ANY-maze overhead cage monitoring	10 min	Distance traveled, mean speed, time in hut, top of hut entries	Baselines and treatments	yes	weeks 0, 2, 3, 4	* 4 ns 2–3		* 4 ns 2–3						* 4 ns 2–3	* 4 ns 2–3			males 12 weeks	[6]
Open field analysis	6 min	distance traveled and number of rears manually tracked	shams and treatments	no	week 8	* 8				* 8								males 21–25 weeks	[21]
Open field analysis	6 min	distance traveled and number of rears manually tracked	naïve and treatments	no	week 8	* 8				* 8								males 22–26 weeks	[10]
Open field testing (Photobeam Activity System)	30 min	ambulation, rearing	shams	yes	weeks 0, 2, 4, 8, 12, 16		* 8–16 ns 2–4			* 8–16 ns 2–4								unknown 10 weeks	[112]
Open field analysis (Accuscan analysis software)	1 h	distance traveled	treatments	no	weeks 6, 14, 24	ns (6–24)												unknown 16 weeks	[111]
Locomotor activity, Overnight (6 h) Ambulation, (Opto-Varames Micro Animal Activity System)	6 h	Total ambulation	baseline	yes	weeks 0, 2, 4, 6, 8		ns 2–8 Males and females look different											F&M 8–12 weeks	[55]
Open field analysis (biobserve)	15 min	track length, average velocity	shams and treatments	no	weeks 2, 8	* 2 ns 8		* 2 ns 8										Females 8 weeks	[40]
RFID tracking of colony movement at jump holes	16 h	cage movement	healthy and different injury types	yes	Weeks 1–12		* 1 ns 2–12											rats males 8–9 weeks	[125]

* Indicates statistical significance at the given weeks; ns signifies non-significance at the given weeks, # indicates number.

**Table A2:** Gait Analysis Results-Mice.

Test Style	Parameters Shown	DMM Compared Against	Baseline	Timepoints Shown	Stance	Swing	Step	Paw Area	Paw Angle	Duty	Stride/Stance/Step Length	Stride/Stance/Step Width/BOS	Velocity	Break	Propel	F/R or L/R	Sex and Age at Time of Injury	Ref
catwalk	paw area, contact area, and swing speed.	Sham and treated	no	weeks 4, 8		* 4–8		paw * 4–8 contact * 4–8									males 10 weeks	[72]
catwalk	print area, max contact area	treated	no	week 8				print * 8 max * 8									males 8 weeks	[79]
catwalk	duty cycle (stance time/(stance time + swing time) * 100%)	baseline and treated	yes	day 0, 1 week 1, 2						* d1,w1 ns w2							males 10–12 weeks	[130]
catwalk	mean intensity, swing speed, swing time, step cycle	treated and contralateral	no	week 12		time * 12 speed * 12	* 12	* 12									males 8 weeks	[131]
catwalk	% mean intensity, % print area, % swing speed, % duty cycle	naked eye vs. microscope DMM	no	weeks 8, 10, 12, 14, 16		* 8 ns 10–12		area * 8–16 mean intensity * 8–14, ns 16		* 8–14 ns 16							males 8 weeks	[67]
catwalk	velocity, paw print area, and duty cycle	naïve and bilateral DMM	yes	weeks 0–16 every week				* 1, 4–16 ns 2–3		* 1 ns 2–16			* 1 ns 2–16				males 10 weeks	[34]
catwalk	RH/LH % print area, RH/LH % Stress, RH/LH % swing speed	Sham and treated	no	weeks 2, 4, 6, 8		* 2–8		* 2–8								* 2–8	males 8 weeks	[96]
catwalk	normalized paw print area %, weight bearing	baseline and treated	yes	weeks 0–12 weekly				* 12 ns 11 weight bearing * 12 ns 11									males 12 weeks	[36]
catwalk	stance phase, swing phase, single stance, duty cycle, swing speed.	Sham and treated	yes	weeks 0, 3, 8, 12, 16	phase * 12–16 ns 3–8 single * 12–16 ns 3–8	phase * 12–16 ns 3–8 speed * 12–16 ns 3–8				* 12–16 ns 3–8							males 9 weeks	[132]
custom gait analysis-free movement down a walkway (MouseWalker)	anterior extreme position (AEP) in parallel, posterior extreme position (PEP) in parallel, stance instability, swing speed, step distance, footprint area imbalance (LH-RH), stance offset, stance duration, and duty factor imbalance.	naïve/baseline	yes	weeks 1, 2, 4, 8, 12	offset * 4 ns 1–2, 8–12 duration * 4 ns 1–2, 8–12	* 4–12 ns 1–2	ns 1–12			* 12 ns 1–8		stance instability * 12 ns 1–8				AEP * 1–12 PEP ns 1–12 LH-RH * 12 ns 1–8	males 12 weeks	[60]
footprint analysis	relative stride length, relative step length, and relative front/rear print length	Sham and treated	no	week 12							stride * 12 stance * 12					* 12	males 12 weeks	[78]
footprint analysis	relative stride length, relative step length, relative front/rear print length	Sham and treated	no	week 12							stride * 8 step * 8					* 8	males 12 weeks	[80]
footprint analysis	front stride length, hindlimb stride length	Sham and treated	no	week 4							front stride * 4 rear stride * 4						males 12 weeks	[81]
footprint analysis	measured stride length, stride width, step distance, and % of right-to-left stride length	Sham and treated	no	weeks 4, 8							distance * 8 ns 4 stride length * 4–8	* 8 ns 4				* 4–8	males 12 weeks	[133]
footprint analysis	relative stride length, relative step length, relative front/rear print length	Sham and treated	no	week 12							stride * 12 step * 12					* 12	males 12 weeks	[15]
footprint analysis	base of support (BOS), right hind paw stride length, and right-left hind paw distance	Sham and treated	no	week 8							* 8	* 8				* 8	males 8 weeks	[134]
footprint analysis	stride length, stance length, sway distance	Sham and treated	no	weeks 2, 4, 6, 8							Stride * 2–8 stance * 2–8	sway * 2–8					males 12 weeks	[16]
footprint analysis	stride length, right-left hind paws distance, and base of support (BOS)	Sham and treated	no	week 8							* 8	* 8				* 8	males 12 weeks	[135]
footprint analysis	stride length	Baseline and sham	yes	weeks 0.4, 1, 2, 4, 8, 12, 16							* 4 ns 1–2, 8–16						males 11 weeks	[51]
footprint analysis	r-l distance, rotation of paws, relative step length, toe spread (TS), intermediate toe spread (ITS, print length (PL).	naïve and sham	no	weeks 4, 6, 8, 10, 12				PL ns 2–12 TS ns 2–12 ITS ns2–12	ns 2–12		ns 2–12					ns 2–12	males 12 weeks	[48]
compulsory treadmill running (DigiGait)18 cm/s	stride length, paw area, and swing time	Sham and treated	no	week 12		* 12		* 12			* 12						males 10 weeks	[14]
compulsory treadmill running (DigiGait) 18 cm/s	stride length, stance width, paw area, and swing time	Sham and treated	no	week 12	* 12	* 12					* 12	* 12					males 10 weeks	[113]
compulsory treadmill running (DigiGait) 17 cm/s	stride length, stance time, paw area, and swing time	Sham and treated	no	week 8	* 8	* 8		* 8			* 8						males 10 weeks	[137]
compulsory treadmill running DigiGait 35 cm/sec	% swing stride, % stance stride, % break stride, % propel stride	baseline and treated	yes	week 0, 2, 3, 4	* 2, 4 ns 3	* 2, 4 ns 3								* 3 ns 2, 4	* 4 ns 2–3		males 12 weeks	[6]
catwalk	mean intensity	All other paws	yes	weeks 0, 2, 4, 6				ns 2–6									females and males 8–12 weeks	[55]
catwalk	log paw intensity	sham	no	weeks 2, 5, 10				* 10 ns 2–5									unknown 10 weeks	[46]
compulsory treadmill running DigiGait 30 cm/sec	break time, paw area, paw angle, midline distance	treated	no	week 16				F * 16	F * 16			F * 16		F * 16			F&M 16 weeks	[138]
custom gait analysis (free walking gait)	velocity, stance time, stride length, step width, temporal symmetry, and spatial symmetry	sham	yes	week 0, 4, 6, 8, 10, 12	* Temporal ns 4–12						* Temporal, 8 ns 4–6, 10–12	* Temporal, 6 ns 4, 8–12	* Temporal ns 4–12			temporal and spatial symmetry ns 4–12	F&M 16 weeks	[136]

* Indicates statistical significance at the given weeks; ns signifies non-significance at the given weeks.
